# Prognostic value of the lymph node ratio for lymph-node-positive breast cancer- is it just a denominator problem?

**DOI:** 10.1186/s40064-015-0865-2

**Published:** 2015-03-11

**Authors:** Upali W Jayasinghe, Nirmala Pathmanathan, Elisabeth Elder, John Boyages

**Affiliations:** Westmead Breast Cancer Institute, Westmead, New South Wales Australia; Faculty of Medicine, University of New South Wales, Sydney, New South Wales Australia; Macquarie University Cancer Institute, Macquarie University, North Ryde, New South Wales Australia

**Keywords:** Lymph node ratio, pN stage, Number of nodes removed, Breast cancer specific survival

## Abstract

**Purpose:**

To examine the prognostic value of lymph node ratio (LNR) for patients with node-positive breast cancer with varying numbers of minimum nodes removed (>5, > 10 and > 15 total node count).

**Methods:**

This study examined the original histopathological reports of 332 node-positive patients treated in the state of New South Wales (NSW), Australia between 1 April 1995 and 30 September 1995. The LNR was defined as the number of positive lymph nodes (LNs) over the total number of LNs removed. The LNR cutoffs were defined as low-risk, 0.01–0.20; intermediate-risk, 0.21– 0.65; and high-risk, LNR >0.65.

**Results:**

The median follow-up was 10.3 years. In multivariate analysis, LNR was an independent predictor of 10-year breast cancer specific survival when > 5 nodes were removed. However, LNR was not an independent predictor when > 15 nodes were removed. In a multivariate analysis the relative risk of death (RR) decreased from 2.20 to 1.05 for intermediate-risk LNR and from 3.07 to 2.64 for high-risk while P values increased from 0.027 to 0.957 for intermediate-risk LNR and 0.018 to 0.322 for high-risk with the number of nodes removed increasing from > 5 to > 15.

**Conclusions:**

Although LNR is important for patients with low node denominators, for patients with macroscopic nodal metastases in several nodes following an axillary dissection who have more than 15 nodes dissected, the oncologist can be satisfied that prognosis, selection of adjuvant chemotherapy and radiotherapy fields can be based on the numerator of the positive nodes.

**Electronic supplementary material:**

The online version of this article (doi:10.1186/s40064-015-0865-2) contains supplementary material, which is available to authorized users.

## Introduction

Axillary lymph node status is one of the most important prognostic factors for breast cancer (Schiffman et al. 2011; Vinh-Hung et al. 2003; Yiangou et al. 1999). Practice has changed from full dissection and/or radiation of the axilla (Fisher et al. 1985) to the use of sentinel node biopsy (SNB) for many patients (Krag et al. 1998). More recently the Z00011 trial (Giuliano et al. 2010; Giuliano et al. 2011; Caudle et al. 2011) was conducted to determine the effects of completion axillary lymph node dissection (ALND) on overall survival in patients with sentinel lymph node (SLN) metastases treated with breast conservation and adjuvant therapy including radiotherapy.

In an era when ALND is performed selectively in patients with a high risk of nodal involvement, the accuracy of the surgical procedure assumes greater importance. There is still debate about what constitutes an adequate axillary dissection in terms of the total number of lymph nodes removed. The current American Joint Committee on Cancer (AJCC) Staging System uses the number of positive LNs and classifies a patient with 1 to 3 positive nodes as having pN1 disease, 4 to 9 positive nodes as pN2 disease, and 10 or more nodes as pN3 (Singletary et al. 2003; Rabban 2010). However, the number of involved lymph nodes is also dependant on the total number of lymph nodes removed and examined, which in turn depends on surgical and pathologic procedures (Vinh-Hung et al. 2009; Wang et al. 2012). Although some studies noted that six nodes were the minimum number of nodes needed to adequately assess the axilla (Katz et al. 2008), it is generally accepted that greater than 10 LNs are required (National Comprehensive Cancer Network 2013). One study found that at least five and 10 nodes are required for node-negative and node-positive patients respectively (Fisher et al. 1981).

Several studies have examined the notion of the LN ratio (LNR), defined as the number of positive LNs over the number of LNs removed, as a potential prognostic factor in breast cancer (Voordeckers et al. 2004; Vinh-Hung et al. 2010; van der Wal et al. 2002; Truong et al. 2010; Danko et al. 2010). Some studies have demonstrated that the LNR was useful in estimating prognosis and should be considered in conjunction with the absolute number of positive lymph nodes in helping guide decisions for breast cancer management (Chagpar et al. 2006; Chagpar et al. 2007).

The extent of axillary dissection varies from centre to centre and country to country and of course, surgeon to surgeon and this has an impact on the risk of loco-regional recurrence and survival and even account for variations in the benefit of post-mastectomy radiation (Boyages & Langlands 1998). An increasing dilemma for clinicians is whether to give patients with 1–3 N+ post-mastectomy radiation therapy, particularly if a patient has had an adequate axillary dissection. In other words, could the survival and loco-regional control benefit of radiation be simply due to under-staging and, does the recent Oxford meta-analysis really mean that every patient with 1–3 nodes positive require post-mastectomy radiation (Zhou et al. 2013). Further, there is ongoing debate, not only about the use of radiation, but also the areas to be treated particularly with respect to the internal mammary chain (Vrana et al. 2013).

When ≤ 10 nodes are removed, there is a greater probability that some patients with ≥ 4 positive nodes will be misclassified as having one to three positive nodes (Fisher et al. 1981). In other words patients are potentially understaged. The median number of nodes dissected was 11 and 7, for Canadian (Ragaz et al. 1997) and Danish (Overgaard et al. 1997) studies respectively which first reported the potential benefit from post-mastectomy radiation. A previous study at our institution examined an average of 25 nodes per case (range, 8–54), and using strict anatomical criteria, the mean number of LN found in axillary level I, II and III were 8 (range, 2–43), 4 (range, 0–19) and 3 (range, 0–11) respectively (Chua et al. 2002).

Several studies have identified that LNR, categorized as low risk (LNR = 0.01–0.20), intermediate risk (LNR = 0.21–0.65) and high risk (LNR > 0.65), was better at predicting breast cancer specific mortality than pN staging as a way to account for the variability in the nodal count (the denominator), for various levels of dissection and number of positive lymph nodes (the numerator) (Vinh-Hung et al. 2009; Vinh-Hung et al. 2010; van der Wal et al. 2002; Danko et al. 2010; Chagpar et al. 2011). In the current TNM classification system, nodal status is based on the absolute number of involved lymph nodes and does not take into account the total number of lymph nodes removed and assumes that all lymph node dissections are the same. Although TNM classification remains the basis of breast cancer staging, LNR may add important prognostic information. Such a ratio, which contains both information regarding the number of positive nodes as well as the denominator as a defacto measure of the adequacy of the dissection, may be superior to the current AJCC staging system in terms of predicting outcomes (Chagpar et al. 2011).

Few studies have examined LNR in patients with varying total numbers of lymph nodes removed. The aim of this study was to examine whether the prognostic value of LNR depends on the number of nodes removed (>5, > 10 and > 15) using data from a statewide population study involving 154 surgeons and 58 pathology practices in Australia’s largest state.

## Method

### Patient selection

The population studied included 848 consecutive patients with invasive breast cancer who had original histological reports and treated in New South Wales (NSW), Australia between 1 April 1995 and 30 September 1995. This was a population-based study collecting data from multiple treatment centers via a notification process involving a central cancer registry. Only patients who had treatment to their breast combined with axillary clearance surgery were included (n = 848).

A total of 154 surgeons performed surgery on 848 patients using 58 different pathology practices. There were 332 nodes positive patients, 515 node-negative and an unknown number of nodes examined (n = 1), (total including nodes negative is 848) during the six months period. Patterns of care to the breast in this cohort have been previously published (Boyages et al. 2010). Patients with < 6 nodes identified (n = 7) or node-negative breast cancer (n = 515) were excluded from the analysis.

The study, therefore included, three hundred and twenty five lymph node-positive cases with > 5 nodes dissected, 262 with > 10 nodes and 149 with > 15 nodes in the analysis. All node-positive patients who had surgery to their breast and axillary clearance surgery with > 5 nodes removed (34% had conservative treatment and 66% mastectomy) were included. One hundred and thirty six had radiation therapy (85% of breast conservation patients and 20% of mastectomy patients) and 181 had chemotherapy. The median number of axillary lymph nodes removed was 15 (range, 6–43). Sentinel node biopsy technique was not used at that time.

### Data analysis

The New South Wales (NSW) Central Cancer Registry (CCR) maintains a register of all cases of cancer diagnosed in NSW since the beginning of 1972. All cases of breast cancer not known to be dead by the NSW CCR were matched against the death records from the NSW Registry of Births, Deaths and Marriages, enhanced by information obtained from the Australian Bureau of Statistics. Ten-year breast cancer survival data of the study group were provided by the NSW CCR. Follow-up time was calculated from the date of first treatment (surgery) to the date of last follow-up or death. The median follow-up was 10.3 years (interquartile range, 5.2–10.5 years).

The LNR was calculated as the total number of positive lymph nodes divided by the total number of lymph nodes found and examined (the denominator). The cohort was then divided into 3 groups based on established LNR cutoffs (Vinh-Hung et al. 2009; Vinh-Hung et al. 2010; van der Wal et al. 2002; Danko et al. 2010; Chagpar et al. 2011). Accordingly, in this study, the LNR cutoffs were defined as low-risk, 0.01–0.20; intermediate-risk, 0.21– 0.65; and high-risk, >0.65. We examined the prognostic value of lymph node ratio (LNR) for patients with node-positive breast cancer with varying numbers of minimum nodes removed (>5, > 10 and > 15 total node count). We also examined the impact of minimum number of lymph nodes removed (all patients, ≤10 or >10 and ≤ 15 or >15) to compare the performance of LNR as prognostic indicators.

Comparison of categories within a characteristic was carried out with the Pearson Chi-square test and, if any of the expected frequencies was less than five, the Fisher exact test was used. A preliminary univariate survival analysis was performed with the Kaplan-Meier method or Cox proportional hazard regression and groups were compared with the log-rank test. Significant or marginally significant predictors in the univariate analysis were included in the multivariate analysis. The independent prognostic effect of LNR was investigated using Cox proportional hazard regression, adjusting for pN stage, age (<40, ≥ 40), pathological tumour size (≤20 mm, or > 20 mm), histological grade (1, 2 or 3), ER status (negative or positive) and chemotherapy (no or yes). Radiation therapy (no or yes) and hormone therapy (no or yes) were not significant in univariate analysis. All statistical analyses were performed using SPSS statistical software (IBM SPSS Statistics version 19, New York, USA). Survival plots were generated using SAS statistical software (version 9.3; SAS Institute, Cary, NC). Our study was approved by the NSW Population and Health Services Research Ethics Committee.

## Results

The mean age of patients at diagnosis was 56 years (range, 25–91 years). The clinical and pathological characteristics of patients and treatment of patients are shown in Table 1. Compared to patients with low-risk LNR, patients with intermediate- or high-risk LNR were more likely to have pathological tumour size over 20 mm (41% vs 67%, P < 0.001), more likely to be grade 3 (54% high-risk vs 36% low-risk, P = 0.02), have pN2 (4-9 N+) disease (28.2% high-risk vs 2.7% low-risk, P < 0.001) or pN3 (≥10 N+) 71.8% high-risk vs 13.1% intermediate-risk, P < 0.001), radiation therapy (33% vs 54%, P = 0.001) and chemotherapy (50% vs >60%, P = 0.007) (Table 1). The relationship with other patient or tumour characteristics did not vary by LNR groups. In particular, LNR was not different by age at diagnosis. Similarly, distribution of ER and hormone therapy over the LNR groups was similar (Table 1).

**Table 1:** **Clinicopathologic characteristics of 325 women with lymph node**-**positive breast cancer according to lymph node ratio (LNR)**

**Characteristic**		**Lymph node ratio**							
		**Low-risk**		**Intermediate-risk**		**High-risk**		**All**	
		**(≤0.20)**		**(>0.20 & ≤0.65)**		**(>0.65)**			
		**(n = 187)**		**(n = 99)**		**(n = 39)**		**(n = 325)**	
	**P value**	**No.**	**(%)**	**No.**	**(%)**	**No.**	**(%)**	**No.**	**(%)**
Age, yrs	0.622								
<40		22	11.8	8	8.1	3	7.7	33	10.2
≥40		165	88.2	91	91.9	36	92.3	292	89.8
Tumour size, mm	<0.001								
0–20		110	58.8	33	33.3	13	33.3	156	48.0
>20		77	41.2	66	66.7	26	66.7	169	52.0
Histological grade	0.063								
1	(0.047)	30	16.0	8	8.1	4	10.3	42	12.9
2		74	39.6	40	40.4	8	20.5	122	37.5
3		68	36.4	43	43.4	21	53.8	132	40.6
Unknown		15	8.0	8	8.1	6	15.4	29	8.9
ER status	0.362								
Negative	(0.869)	15	8.0	7	7.1	2	5.1	24	7.4
Positive		48	25.7	16	16.2	7	17.9	71	21.8
Unknown		124	66.3	76	76.8	30	76.9	230	70.8
No. of nodes removed	0.249								
6–10		30	16.0	26	26.3	7	17.9	63	19.4
11–15		64	34.2	35	35.4	14	35.9	113	34.8
>15		93	49.7	38	38.4	18	46.2	149	45.8
pN stage	<0.001								
pN1 (1–3)		182	97.3	28	28.3	0	0.0	210	64.6
pN2 (4–9)		5	2.7	58	58.6	11	28.2	74	22.8
pN3 (≥10)		0	0.0	13	13.1	28	71.8	41	12.6
Radiation therapy	0.001								
No		125	66.8	45	45.9	18	46.2	188	58.0
Yes		62	33.2	53	54.1	21	53.8	136	42.0
Chemotherapy	0.007								
No		92	50.3	30	30.9	15	39.5	137	43.1
Yes		91	49.7	67	69.1	23	60.5	181	56.9
Hormone therapy	0.278								
No		79	42.2	33	33.3	13	33.3	125	38.5
Yes		108	57.8	66	66.7	26	66.7	200	61.5

P values are for comparison of categories of each variable by lymph node ratio using the Pearson chi-square test or the Fisher exact test. For characteristics with an unknown category the P values without unknown category are shown within parentheses. Patients with > 5 nodes removed included in the table.

Missing values: Radiation therapy = 1, Chemotherapy = 7.

**Figure 1:**
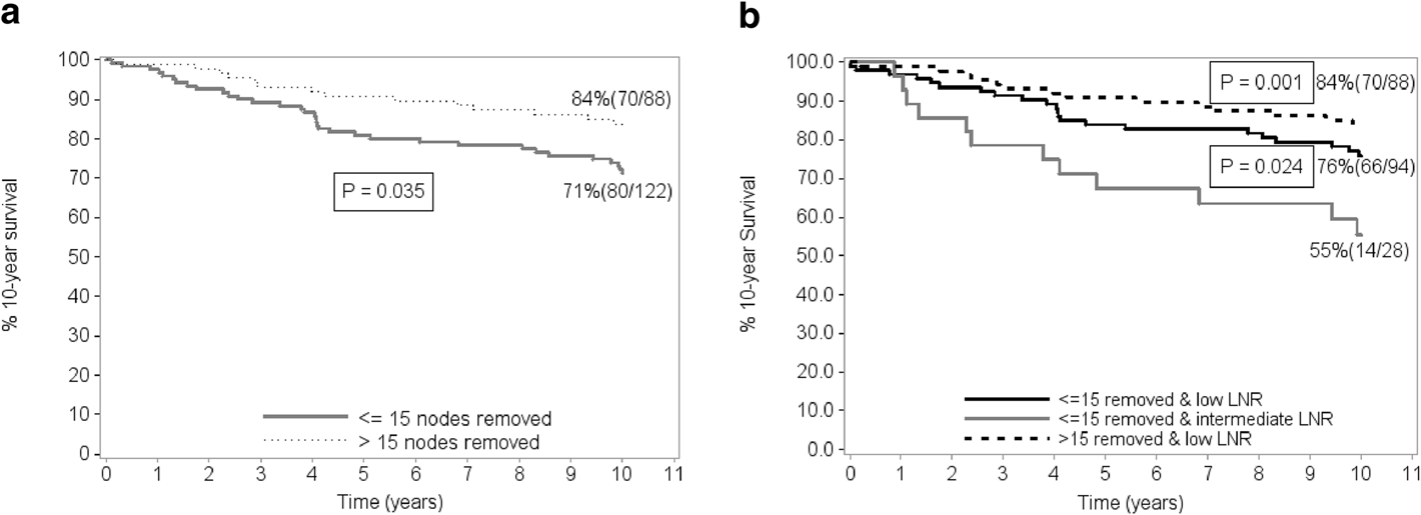
**Kaplan-Meier breast cancer specific survival estimates according to the number of nodes removed. (a)** shows overall survival for pN1 (1–3) stage. Numbers in parentheses indicate total number and number at risk. **(b)** shows overall survival for pN1 (1–3) stage by LNR. Numbers in parentheses indicate total number and number at risk. There were no cases from high risk LNR for ≤ 15 nodes removed. All cases for pN1 stage were low-risk LNR for > 15 nodes removed. P values are from the log rank-test with intermediate LNR as the reference.

At a median follow-up of 10.3 years, 37 of 187 patients (19.8%) with low-risk LNR have died of breast cancer compared to 46 of 99 (46.5%) with intermediate-risk and 23 of 39 (59.0%) with high-risk LNR (P < 0.001). Table 2 shows two scenarios using a denominator of 10 or 15 nodes identified in the final pathology report. Although for patients with 1–3 nodes positive, increasing LNR was associated with increasing mortality when ≤ 10 (low-risk: 16.7%; intermediate risk: 41.2%) or >10 nodes were removed (low-risk: 19.7%; intermediate risk: 45.5%), LNR lost its significance (low risk: 15.9% and no other risk categories) when more than 15 nodes were dissected. Figure 1a shows, that for patients with pN1 (1-3 N+) disease, patients with more than 15 nodes dissected had a 10-year breast cancer-specific survival of 84% compared to 71% when up to 15 nodes were dissected (p = 0.035). Figure 1b shows the corresponding 10-year breast cancer specific survival rates for all possible combinations of LNR for patients with pN1 (1-3 N+). The best 10-year breast cancer specific survival rate was 84% for a low LNR and high nodal denominator (>15 nodes identified). In contrast, for pN1 patients with up to 15 nodes dissected and a low LNR the 10-year survival rate was 76% but dropped to 55% for an intermediate LNR (p = 0.024). There were no patients with a high LNR in the pN1 subgroup.

**Table 2:** **Percentage of breast cancer deaths by LNR and pN stage for varying total numbers of nodes removed**

**pN stage**	**Nodes removed**	**Number of deaths/total (%) for LNR**			
		**Low-risk**	**Intermediate-risk**	**High-risk**	**All**
		**(≤0.20)**	**(>0.20 & ≤0.65)**	**(>0.65)**	
***Nodes removed*** **≤** ***10 and*** **>** ***10***					
pN1 (1–3)	≤10	5/30 (16.7)	7/17 (41.2)	*	12/47 (25.5)
	>10	30/152 (19.7)	5/11 (45.5)	*	35/163 (21.5)
pN2 (4–9)	≤ 10	*	2/9 (22.2)	3/7 (42.9)	5/16 (31.3)
	> 10	2/5 (40.0)	24/49 (49.0)	2/4 (50.0)	28/58 (48.3)
pN3 (≥10)	≤10	*	*	*	*
	>10	*	8/13 (61.5)	18/28 (64.3)	26/41 (63.4)
***Nodes removed*** **≤** ***15 and*** **>** ***15***					
pN1 (1–3)	≤15	21/94 (22.3)	12/28 (42.9)	*	33/122 (27.0)
	>15	14/88 (15.9)	*	*	14/88 (15.9)
pN2 (4–9)	≤ 15	*	15/33 (45.5)	5/11 (45.5)	20/44 (45.5)
	> 15	2/5 (40.0)	11/25 (44.0)	*	13/30 (43.3)
pN3 (≥10)	≤15	*	*	5/10 (50.0)	5/10 (50.0)
	>15	*	8/13 (61.5)	13/18 (72.2)	21/31 (67.7)

*no cases from this category.

For patients with 10 or more positive nodes (pN3) mortality was over 60% irrespective of LNR in a clinical setting where most oncologists would advise post-mastectomy RT. For pN2 disease (4–9 nodes positive), LNR only made a difference in the setting where ≤ 10 nodes were dissected again implying that if the denominator increased to 15 or more, more patients would have had disease that would have been pN3 (≥10 N+) (Table 2).

We also analyzed LNR by number of nodes dissected (all patients or ≤ 15 or >15) (Table 3). Although increasing LNR was a predictor of breast cancer mortality for all patients by univariate or multivariate analysis, it once again lost its significance when more than 15 nodes were identified (Table 3). In a multivariate analysis the relative risk of death (RR) decreased from 2.20 to 1.05 for intermediate-risk LNR and from 3.07 to 2.64 for high-risk while P values increased from 0.027 to 0.957 for intermediate-risk LNR and 0.018 to 0.322 for high-risk with the number of nodes removed increasing from > 5 to > 15.

**Table 3:** **Results of univariate and multivariate survival analysis at 10-year follow up**

	**All patients (n = 318)**				**lymph nodes removed ≤15 (n = 172)**			**lymph nodes removed > 15 (n = 146)**		
	**Univariate cox regression analysis**		**Multivariate cox regression analysis**		**Multivariate analysis cox regression analysis**			**Multivariate analysis cox regression analysis**		
**Factor**	**RR (95% CI)**	**P**	**RR (95% CI)**	**P**	**No.**	**RR (95% CI)**	**P**	**No.**	**RR (95% CI)**	**P**
LNR										
≤0.20	1.00		1.00		92	1.00		93	1	
>0.2–0.65	2.91 (1.90–4.47)	<.001	2.20 (1.09–4.41)	0*.*027	59	1.94 (0.91–4.12)	0*.*085	38	1.05 (0.20–5.54)	0*.*957
>0.65	4.31 (2.56–7.25)	<.001	3.07 (1.21–7.80)	0*.*018	21	2.03 (0.56–7.33)	0*.*282	18	2.64 (0.39–18.04)	0*.*322
pN stage										
pN1 (1–3)	1.00		1.00		118	1.00		88	1	
pN2 (4–9)	2.52 (1.62–3.91)	<.001	1.28 (0.63–2.60)	0.500	44	1.15 (0.51–2.60)	0.745	30	4.31 (0.88–21.20)	0.073
pN3 (≥10)	3.85 (2.38–6.21)	<.001	1.31 (0.56–3.08)	0.533	10	1.03 (0.23–4.64)	0.970	31	5.03 (0.86–29.45)	0.073
Age at diagnosis										
<40	1.68 (0.99–2.86)	0.056	1.82 (1.03–3.22)	0.038	15	1.49 (0.61–3.66)	0.384	18	3.43 (1.53–7.69)	0.003
≥40	1.00		1.00		157	1.00		131	1.00	
Tumour size, mm										
1–20	1.00		1.00		83	1.00		72	1.00	
>20	2.25 (1.51–3.37)	<.001	1.52 (0.99–2.35)	0.058	89	1.95 (1.08–3.51)	0.027	77	0.76 (0.35–1.64)	0.487
Histological grade										
1	1.00		1.00		22	1.00		20	1.00	
2	2.16 (0.97–4.83)	0.060	1.84 (0.81–4.18)	0.143	58	2.18 (0.63–7.60)	0.221	62	1.79 (0.57–5.60)	0.318
3	3.21 (1.46–7.05)	0.004	2.21 (0.98–4.95)	0.055	77	3.73 (1.13–12.32)	0.031	53	1.40 (0.42–4.60)	0.584
Unknown	1.35 (0.45–4.02)	0.590	1.03 (0.34–3.11)	0.958	15	1.51 (0.30–7.60)	0.619	14	0.73 (0.15–3.69)	0.706
ER										
Negative	1.00		1.00		11	1.00		13	1.00	
Positive	0.37 (0.18–0.77)	0.008	0.49 (0.23–1.06)	0.071	36	0.59 (0.19–1.87)	0.367	35	0.20 (0.06–0.65)	0.007
Unknown	0.59 (0.32–1.09)	0.091	0.60 (0.32–1.13)	0.115	125	0.76 (0.29–1.95)	0.562	101	0.26 (0.10–0.66)	0.005
Chemotherapy										
No	1.00		1.00		96	1.00		61	1.00	
Yes	1.58 (1.05–2.37)	0.028	1.04 (0.67–1.61)	0.860	76	1.24 (0.69–2.22)	0.480	85	0.61 (0.29–1.30)	0.202

RR: Relative risk of dying; 95% CI: 95% confidence interval of relative risk.

P value is for comparison of each category with the reference category.

Number in each category is shown in Table 1. Seven missing values for chemotherapy.

Radiation therapy (P = 0.587) and hormone therapy (P = 0.762) are not significant in univariate analysis and not included in multivariate analysis.

## Discussion

LNR has been widely demonstrated to be a useful alternative to predict survival in many cancers including lung cancer (Matsuguma et al. 2012; Jonnalagadda et al. 2011), colon cancer (Greenberg et al. 2011; Berger et al. 2005), pancreas (Berger et al. 2004), bladder (Herr 2003), gastric cancer (Tong et al. 2011; Hong et al. 2011) and particular breast cancer (Schiffman et al. 2011; Vinh-Hung et al. 2009; Danko et al. 2010; Chagpar et al. 2011). In contrast to many other studies, we found that LNR was not important if an adequate axillary dissection was performed. We hypothesise that LNR is a problem of the denominator. When patients with breast cancer have a nodal dissection identifying more than 15 nodes, LNR loses its significance.

Woodward et al. (2006) conducted a systematic review based on 24 studies published from 1994 to 2005 totaling 32,299 patients supporting the greater prognostic value of LNR compared to number of involved nodes. Table 4 shows the prognostic value of LNR for more recent studies published since 2008 and these studies again confirmed the superiority of LNR (Schiffman et al. 2011; Vinh-Hung et al. 2009; Danko et al. 2010; Chagpar et al. 2011; Saxena et al. 2012; Truong et al. 2008; Li et al. 2012; Duraker et al. 2013; Dings et al. 2013). However, in these studies all patients were included in the analysis and the denominator of LNR (number of nodes removed) starting from one node found leading to high LNR’s when the denominator was low and reduced survival simply because of under-staging of the axilla. Similar to our study all of these studies but one examined the prognostic value of LNR after adjustment for pN stage. We found that LNR was no longer significant when the denominator was >10 (Tables 2 and 3) and particularly when >15 nodes were identified (Table 3).

**Table 4:** **Recent studies (2008–2013) of ratios of involved nodes in breast cancer**

**Study**	**Years**	**No. of patients**	Selection of patients*	**LNR cut-off or groups**	**Follow-up period**	**Outcome and Prognostic role of LNR in multivariate analysis**	**Nodes removed median (range)**	**Population/Institution study**
Truong et al. 2008	1988–1997	4571	T_1_–T_2_	≤0.25,	14 years	BCCS and OS significant	15 (1–50)	Population
			Node-positive	>0.25				
Vinh-Hung et al. 2009	1980-2004	1829	T_1_–T_3_	1. LNR, continuous.	10 years	BCSS significant for continuous and categorical LNR	14 (1–X)	Population
			Node-positive	2. ≤0.20,0.21-0.65, >0.65				
Danko et al. 2010	1985-2005	1788	T_1_–T_3_	≤0.20,0.21-0.65, >0.65	10 years	DFS and OS significant	17 (1–57)	Institution
			Node-positive					
Schiffman et al. 2011	1996-2007	556	T_1_–T_3_	≤0.20,0.21-0.65, >0.65	5 years	DFS and OS significant	3 (1–52)	Population
			Node-positive					
Chagpar et al. 2011	1956-1982	319	T_1_–T_3_	≤0.20,0.21-0.65, >0.65	40 years	OS significant	13 (1–48)	Institution
			Node-positive					
Li et al. 2012	1998-2000	127	T1–T4	LNR, continuous.	10.9 years	OS significant	X	Institution
			Node-positive					
Saxena et al. 2012	1990-2007	1589	T1–T3	≤ .20,0.21-0.65, >0.65	5 years	OS significant	15 (1–X)	Institution
			Node-positive					
Duraker et al. 2013	1993–2002	2151	T1–T3	≤ .20,0.21-0.65, >0.65	7 years	DFS significant	14 (1–46)	Institution
			Node-positive					
Dings et al. 2013	1999-2005	25315	T1–T3	≤ .20,0.21-0.65, >0.65	10 years	OS significant	14 (1–90)	Population
			Node-positive					
Current series	1995	325	T_1_–T_3_	≤0.20,0.21-0.65, >0.65	10 years	1. BCSS not significant if > 15 nodes removed.	15 (6–43)	Population
			Node-positive					
						2. BCSS significant for all patients.		

Notes: *Node-positive patients were considered if possible.

BCSS-breast cancer specific survival. DFS-disease-free survival.

OS-overall survival from any cause or cause not mentioned. X–unknown.

The likelihood of finding positive nodes in the axilla increases with the number of nodes removed, similarly the likelihood of having residual disease in the axilla decreases with more extensive dissection (Krag & Single 2003; McMasters 2003). In this study, LNR appeared to be important only for patients who had <10 nodes identified by the pathologist after an axillary dissection. Once the level of dissection of the axilla increased to 10 or more nodes and particularly more than 15 nodes, LNR was not significant in a multivariate analysis.

The study demonstrates that the LNR predicted survival more accurately than pN stage for all patients and when both ≤ 15 or > 15 nodes removed were included in the analysis (Table 3) consistent with other studies in which all patients with varying node counts were included in the analysis (Vinh-Hung et al. 2009; Voordeckers et al. 2004; Truong et al. 2005; Ahn et al. 2011). In our series, almost half of our patients (45.9%) had a denominator of >15 nodes removed. In a large study from the Netherlands, 37.4% of patients had > 15 nodes removed (Dings et al. 2013). In a multivariate analysis relative risk of dying (RR) increased from 1.28 to 4.31 for pN2 (4-9 N+) and from 1.31 to 5.03 for pN3 (≥10 N+) and P values decreased from 0.5 to 0.073 for pN2 and 0.53 to 0.073 for pN3 with the number of nodes removed increasing from > 5 to > 15 (Table 3).

It is likely that some patients from the intermediate-risk LNR were understaged as pN1 stage as a result of the small number of nodes removed (Table 2). Further, this conclusion is supported by the number of positive nodes in the intermediate-risk LNR (median = 3, range, 2–3) for groups where ≤ 10 or ≤ 15 nodes removed. When ≤ 15 nodes are removed, there is a greater probability that some patients with ≥ 4 positive nodes will be misclassified as having one to three positive nodes. This is in contrast to those patients where > 15 nodes are removed where all pN1 patients were from the low-risk LNR.

Most studies of LNR in breast cancer are single-institution studies that create their own LNR groupings based on their institution’s data (Katz et al. 2008; Voordeckers et al. 2004; van der Wal et al. 2002). The number of lymph nodes removed and examined is highly dependent on surgical technique and expertise, the institution’s protocol for identifying lymph nodes and the pathologists’ experience (Schaapveld et al. 2004). Our study is one of the few population based studies that examined the LNR risk categories established by previous studies (Vinh-Hung et al. 2009; Vinh-Hung et al. 2010; van der Wal et al. 2002; Danko et al. 2010; Chagpar et al. 2011) and involved a large number of surgeons with varying expertise in performing axillary clearance. The duration of follow-up in our study is longer than that in many other studies (median = 10.3 years), which increases the ability to assess the prognostic value of the variables being investigated. Other advantages of our study are the larger median number of nodes removed (n = 15) and accurate follow up data with breast cancer specific survival based on information received from a centralised cancer registry which included information on cause of death.

Some authors argue that the additional information from performance of completion ALND after positive SLN biopsy may benefit patients by guiding decisions about adjuvant chemotherapy. For the approximately one-half of patients in whom there is residual nodal disease, it is also argued that complete ALND can influence survival via local-regional control of the axilla, thereby eliminating a potential site of recurrent disease and, ultimately, a source for distant disease (Straver et al. 2010; Kothari et al. 2012; Sosa et al. 1998). The standard of care for breast cancer patients with sentinel lymph node (SLN) metastases includes complete ALND (Van Zee et al. 2003). The Z-0011 trial has advocated that ALND is not necessary if < 3 nodes are involved (Giuliano et al. 2010). Giuliano et al. (2011) summarized total nodal involvement in ALND and SLND alone groups, 21.0% of patients undergoing ALND had 3 or more involved nodes compared with 3.7% undergoing SLND alone (Giuliano et al. 2011). Completion ALND quantifies total nodal burden, defines prognosis and can determine adjuvant treatments in addition to maximising local disease control (Kothari et al. 2012). However, the Z-0011 data has questioned the need for completion ALND for early node-positive breast cancer treated with conservation, RT and optimal systemic therapy. Thus our data, need to be placed into context of changes in recent strategy particularly for older patients with smaller primary tumors and low sentinel node positivity particularly for luminal A tumors where RT to the axilla is also given (Jagsi et al. 2014). However, for patients with macroscopic nodal metastases in several nodes following an axillary dissection who have more than 15 nodes dissected, the oncologist can be satisfied that prognosis, selection of adjuvant chemotherapy and radiotherapy fields can be based on the numerator of the positive nodes. When patients have low node denominators and higher numerators, our data suggests that LNR is important and treatment selection may need to be intensified to take into account potential understaging of the axilla.

### Ethical standards

Research conducted for the purpose of this manuscript complies with the current laws of Australia.
